# The role of the nervous system in aging and longevity

**DOI:** 10.3389/fgene.2013.00124

**Published:** 2013-06-27

**Authors:** Joy Alcedo, Thomas Flatt, Elena G. Pasyukova

**Affiliations:** ^1^Department of Biological Sciences, Wayne State UniversityDetroit, MI, USA; ^2^Department of Ecology and Evolution, University of LausanneLausanne, Switzerland; ^3^Laboratory of Genome Variation, Institute of Molecular Genetics, Russian Academy of SciencesMoscow, Russia

The connections between the nervous system, aging and longevity are manifold and profound. On the one hand, the nervous system plays an important role in processing complex information from the environment, which has a major influence on an animal's aging and longevity. Accordingly, environmental signals are received and integrated by this organ system, leading to diverse physiological outputs that can have pervasive effects on homeostasis and lifespan. Thus, an animal's nervous system not only controls its homeostatic responses but can also alter its lifespan and aging process. On the other hand, similar to the feedback regulation that characterizes homeostatic mechanisms, aging also impacts the functional state of the nervous system, as exemplified, for instance, by the prevalence of age-associated neurodegenerative diseases.

The lead review (Alcedo et al., 2013) in this collection of articles introduces different aspects of neuronal inputs and outputs of signaling pathways that affect homeostasis, and consequently longevity and aging. A number of these inputs, which can be detected by sensory neurons acting at the interface between an animal's external and internal environments, have been found to either shorten or lengthen lifespan. Jeong et al. (2012) describe more explicitly how gustatory, olfactory and thermosensory cues affect invertebrate lifespan and the possible implications on mammalian aging. In mammals, these sensory cues likely modulate hypothalamic function and the neuroendocrine systems required for maintaining homeostasis [reviewed in Alcedo et al. (2010, 2013); Jeong et al. (2012)]. Consistent with this notion, Bartfai and Conti (2012) discuss how nutrient signals act on heat-sensing hypothalamic neurons to regulate energy expenditure by maintaining mammalian core body temperature, which has been previously shown to affect lifespan (Conti et al., 2006).

In addition to nutritional and temperature cues, the nervous system can sense other environmental cues and stressors (Alcedo et al., 2013). Iranon and Miller (2012) focus on one such stressor—i.e., low oxygen availability, which compromises many important physiological processes; in their paper, the authors review the mechanisms animals use to maintain oxygen homeostasis in response to hypoxia. In contrast, Kagias et al. (2012) elaborate on different types of stressors and the neuronal responses that these elicit. Besides extrinsic environmental factors, they also discuss how the processes of development and aging generate intrinsic stress (Kagias et al., 2012).

Neuronal inputs are integrated by neural circuits, which can then lead to longevity-modulating outputs. Because of the central role that calcium signaling plays in processing neural information, Nikoletopoulou and Tavernarakis (2012) highlight the importance of maintaining calcium homeostasis. They discuss how loss of calcium homeostasis increases the risk for neurodegenerative diseases and how aging itself can impair this homeostasis (Nikoletopoulou and Tavernarakis, 2012). Calcium homeostasis requires the coordinated function of different organelles, including that of the mitochondria (Nikoletopoulou and Tavernarakis, 2012). Troulinaki and Bano (2012) further underscores the involvement of mitochondria in known longevity pathways and their dual role in aging and neurodegeneration. They review how the decline of mitochondrial activity significantly contributes to age-related impairment of neural circuits (Troulinaki and Bano, 2012).

Consistent with this idea, several reviews discuss evidence suggesting that impaired, aging neurons can modulate the functional outputs of the nervous system, such as protein homeostasis (David, 2012), learning, memory (Stein and Murphy, 2012), and emotional state (McKinney et al., 2012). The review of David (2012) focuses on the role of protein aggregation and protein-quality control in the aging brain. Of note, several reviews discuss how neuronal signaling upon mitochondrial dysfunction, in addition to other stimuli, plays a role in coordinating the mitochondrial unfolded protein response, which in turn affects protein misfolding and polyglutamine aggregation in non-neuronal tissues (David, 2012; Kagias et al., 2012; Alcedo et al., 2013).

McKinney et al. (2012) and Stein and Murphy (2012) present other mechanisms through which aging neurons affect the rate of organismal senescence. The impact of neuronal aging on cognitive and psychological states can be observed through consistent and specific age-dependent gene expression changes in the brain. The review by McKinney et al. (2012) also supports the important notion that naturally occurring individual variation in the rates of gene expression changes during brain aging can determine the onset of senescence and of developing age-related brain disorders. Last but not least, the paper by Huffman (2012) reviews the intimate links between the regulation of development and aging by discussing how patterns of neocortical gene expression and neocortical sensory-motor axonal connections develop and change throughout the lifespan of the animal and how they affect aging.

Collectively, the reviews in this Special Topic provide ample evidence from recent literature that show how aging is modulated by a complex interplay between the environment, genes, signaling networks and tissues. In particular, they highlight the key role in aging and longevity played by the nervous system, which is *the* central integrator of information from both the external environment and the inner “milieu” intrinsic to the organism. In discussing the state-of-the-art, these papers also illustrate key areas for future work. For example, our current understanding of the sensory perception of environmental cues and the signals that integrate and process these cues in affecting lifespan and aging is still very limited. Moreover, major and unresolved questions remain about the mechanistic relationship between the neuronal regulation of lifespan and the senescence of neuronal and cognitive function. Finally, we still lack essential information on the evolutionary conservation of the neuronal inputs and outputs of longevity and aging. Such information might prove to be extremely helpful in generating therapeutic/pharmacological interventions in the future. As is clearly demonstrated by the review articles in this Special Topic, the neurobiology of aging is a rapidly developing field; at the same time, numerous difficult problems remain to be solved in future work, making this vibrant field a major frontier in aging research.
